# A Compendium of *in vitro* Germination Media for Pollen Research

**DOI:** 10.3389/fpls.2021.709945

**Published:** 2021-07-09

**Authors:** Donam Tushabe, Sergey Rosbakh

**Affiliations:** Ecology and Conservation Biology, Institute of Plant Sciences, University of Regensburg, Regensburg, Germany

**Keywords:** cookbook, experiment, medium, *in vitro*, pollen

## Abstract

The correct choice of *in vitro* pollen germination media (PGM) is crucial in basic and applied pollen research. However, the methodological gaps (e.g., strong focus of current research on model species and cultivated plants along with the lack of general rules for developing a PGM) makes experimenting with pollen difficult. We closed these gaps by compiling a compendium of optimized *in vitro* PGM recipes from more than 1800 articles published in English, German, and Russian from 1926 to 2019. The compendium includes 1572 PGM recipes successfully used to germinate pollen grains or produce pollen tubes in 816 species representing 412 genera and 114 families (both monocots and dicots). Among the 110 components recorded from the different PGM recipes, sucrose (89% of species), H_3_BO_3_ (77%), Ca^2+^ (59%), Mg^2+^ (44%), and K^+^ (39%) were the most commonly used PGM components. PGM pH was reported in 35% of all studies reviewed. Also, we identified some general rules for creating PGM for various groups of species differing in area of research (wild and cultivated species), phylogenetic relatedness (angiosperms vs. gymnosperms, dicots vs. monocots), pollen physiology (bi- and tri-cellular), biochemistry (starchy vs. starchless pollen grains), and stigma properties (dry vs. wet), and compared the component requirements. Sucrose, calcium, and magnesium concentrations were significantly different across most categories indicating that pollen sensitivity to sugar and mineral requirements in PGM is highly group-specific and should be accounted for when composing new PGM. This compendium is an important data resource on PGM and can facilitate future pollen research.

## Introduction

Pollen, the male gametophyte, is an evolutionary development in higher plants that ensures successful genetic exchange, establishment, and survival of the species (Ashman et al., 2004; Pacini and Dolferus, 2016). Because of their crucial role in successful seed development (Shivanna and Rangaswamy, 1992; Dafni and Firmage, 2000; Rosbakh et al., 2018), pollen germination (PG), and pollen tube growth (PTG) have been in the focus of many studies ranging from research on physiological and biochemical aspects of these processes (Taylor and Hepler, 1997; Wang et al., 2010; Williams and Reese, 2019) to large-scale screenings of pollen abiotic stress-tolerance (Kakani et al., 2005; Rosbakh et al., 2018). Additionally, pollen is an excellent model system for studying a number of basic processes including plant cell growth, cell wall synthesis, intracellular transport, and cell-cell interaction (Johnson-Brousseau and McCormick, 2004; Boavida and McCormick, 2007). That is why pollen remains one of the most attractive objects in plant research.

In basic and applied research, pollen functioning has been studied with the help of two approaches, *in vivo* and *in vitro*. *In vivo* studies are carried out directly at the stigmatic surface in the natural state, while *in vitro* approaches rely on a culture medium that simulates conditions of the style-stigma (Rodriguez-Enriquez et al., 2013). The advantage of the *in vivo* methods is that they consider all natural conditions pollen grains experience on stigma (Dawkins and Owens, 1993; Albert et al., 2018). However, such methods have sometimes proved difficult (Shivanna and Rangaswamy, 1992). Partly, this is due to the involvement of the pistillate tissue that interacts with the growing pollen tubes thereby affecting physiological and biochemical investigations (Shivanna and Rangaswamy, 1992; Zheng et al., 2019; Xu et al., 2020). Moreover, the complex and labor-intensive nature of the *in vivo* approach (e.g., maintenance of pistil tissue viability and post-experimental sample processing) limits its applicability in large-scale research, such as breeding programs (Kakani et al., 2005) or multispecies ecological screenings (Rosbakh et al., 2018). The comparatively technically simple *in vitro* approach, which is based on the ability of pollen to germinate and grow without the pistillate tissue, solves this problem making it possible to conduct comprehensive pollen research (Taylor and Hepler, 1997). Although being sometimes criticized for inaccurate replication of the biological context (Rodriguez-Enriquez et al., 2013), the *in vitro* approach is generally preferred because it provides results comparable to *in vivo* studies (Sulusoglu and Cavusoglu, 2014; Jayaprakash, 2018; Luo et al., 2020). It is also easier to detect alterations in PG or PTG performance using *in vitro* approaches (Procissi et al., 2003; Steinebrunner et al., 2003; Cole et al., 2005; Hashida et al., 2007) since the parameters can easily be tested for defects which is difficult to perform *in vivo*. Notably, *in vitro* PG rates are considered the best estimate of pollen viability *in vivo* (Shivanna et al., 1991; Stone et al., 1995).

Typically, an *in vitro* PG protocol includes cultivating of fresh or stored pollen grains in/on a germination media contained within a hanging drop/well or on a membrane support (Conner, 2011; Soares et al., 2013; Jayaprakash, 2018). In all such protocols, the correct choice of pollen germination media (PGM) remains the most important part, as pollen is highly sensitive to the PGM composition (Dafni et al., 2005). To begin with, pollen of several species requires either liquid (Hoffmann et al., 1990; Golan-Goldhirsh et al., 1991; Montaner et al., 2003) or solid (e.g., with addition of agar) medium (Shivanna and Sawhney, 1995; Jayaprakash, 2018) to germinate, while others can germinate in/on both solid and liquid media (Bilderback, 1981; Boavida and McCormick, 2007). Additionally, most pollen grains need a carbohydrate source to germinate successfully, and sucrose solution is generally used (Montaner et al., 2003; Silva et al., 2016; Lagera et al., 2017). In some cases, other sugars and sugar derivatives such as lactose, maltose, raffinose, and fructose among others, are also used (Shaoling et al., 2005; Hirsche et al., 2017; Lagera et al., 2017; Impe et al., 2019). Furthermore, several inorganic compounds affect *in vitro* PG with boron being one of the most important element for most species (Brewbaker and Kwack, 1963; Wang et al., 2003; Yao and Zhao, 2004; Fang et al., 2016). Besides boron, minerals such as calcium, magnesium, potassium are also known to have stimulatory effects on PG (Brewbaker and Kwack, 1963; Čapková-Balatková et al., 1980; Song et al., 2009; Biswas and Mondal, 2014; Jayaprakash, 2018). The different compounds in the medium affect the pH that must therefore be adjusted to species-specific values, to allow for optimal conditions for pollen to germinate (Tupý and Řhová, 1984; Fricker et al., 1997; Fan, 2001). Trisaminomethane (Tris) and 2-ethanesulfonic acid (MES) belong to the buffers that has been often used to maintain a constant pH in the medium (Tupý and Řhová, 1984; Holdaway-Clarke et al., 2003). Further, several other substances such as hormones, vitamins, buffers, proteins, lipids, antibiotics, enzymes, plant, and animal extracts are sometimes added to increase the percentage of PG and to accelerate the rate of PTG (Vasil, 1960; de Bruyn, 1966; Boavida and McCormick, 2007; Jayaprakash, 2018). Finally, different plant groups are also suggested to have different PGM requirements (e.g., plants with binucleate vs. trinucleate pollen grains (Hoekstra, 1979; Bergamini and Mulcahy, 1988; Zhang et al., 1997; Gibernau et al., 2003); those with dry vs. wet stigmas (Boavida and McCormick, 2007; Rodriguez-Enriquez et al., 2013); angiosperms vs. gymnosperms (Paoletti and Bellani, 1990); monocots vs. dicots (Jayaprakash, 2018); and in plants with starchy vs. starchless pollen grains (Bellani et al., 1985; Franchi et al., 2007).

Despite the fact that several PGM are widely available (Brewbaker and Kwack, 1963; Hong-Qi and Croes, 1982; Roberts et al., 1983; Rodriguez-Enriquez et al., 2013), experimentation on PG *in vitro* is still challenged by several problems. Firstly, information on PGM requirements for a species in question is usually extremely scattered in works published in very different journals and/or years. Secondly, although numerous protocols describe methods to induce PG, they are not applicable to all species as they are strongly biased either to model species, such as *Arabidopsis thaliana* (Boavida and McCormick, 2007; Rodriguez-Enriquez et al., 2013) or domesticated plant species, their cultivars and wild relatives (Roberts et al., 1983; Cheng and Mcomb, 1992; Jayaprakash et al., 2018). In contrast, PG studies for any given wild species are limited (Mortenson et al., 1964; Fernando et al., 2001; Sorkheh et al., 2011). Thirdly, it is not clear whether the available protocols can be generalized to create some general rules for developing a PGM for a single species or various species groups especially those that have not yet been studied.

Here, we close these gaps by compiling a compendium of *in vitro* PGM recipes available in the published literature. Specifically, we first provide a list of optimized media successfully used to germinate pollen grains and/or produce pollen tubes in different species. In addition, we identify the key PGM components required for various groups of species differing in area of research (wild and cultivated species), phylogenetic relatedness (angiosperms vs. gymnosperms, dicots vs. monocots), pollen physiology (bi- and tri-cellular), biochemistry (starchy vs. starchless pollen grains), and stigma properties (dry vs. wet) that will help to create and/or optimize PGM recipes for the species, for which such information is not available.

## Materials and Methods

To extract available information on PGM composition, we first reviewed all studies published from 1926 to 2019 that included keywords ‘‘pollen’’, ‘‘germination,’’ and ‘‘media’’ either in the title or abstract. The literature was searched using the Web of Science database^1^ with the searched databases including, “Web of Science Core Collection,” “KCI-Korean journal database,” “Medline,” “Russian science citation index,” and “SciELO Citation.” Only publications in English, Russian, and German were considered. The search resulted in 1800 studies.

In the second step, we sorted out the publications that were accessible in a digital form and/or contained information about PGM composition with an estimate of its efficacy to stimulate PG or PTG in the abstract or full text. From each of the 675 studies that fulfilled the selection criteria, we extracted the author name(s), year of publication, full literature reference and whether the publication was available in digital form. For each species/variety studied in the selected publications, we further extracted information on the PGM composition including the ingredients used and their concentrations. When several PGMs were used in a publication, only PGM reported to be most effective, i.e., resulting in maximum PG, longest pollen tubes, or minimum pollen bursting obtained, were extracted. If component concentrations of effective PGMs were given as a range, average values of such ranges were considered.

Additionally, we included unpublished data on PGM composition for 104 Central European plant species collected during PG studies at the University of Regensburg from 2014 to 2020 (S. Rosbakh unpublished).

### Data Analysis

For the statistical analysis, all entries in the data set were standardized by recalculating sucrose and agar concentrations to percentages and the rest of the ingredient concentrations to millimolar (mM). The species taxonomy was standardized against the “Plant List” (2013).

In order to infer group-specific concentrations of PGM components, we classified all species present in the data set into several categories: (1) wild vs. cultivated species, (2) angiosperms vs. gymnosperms, (3) dicots vs. monocots plants, (4) bi- vs. tri-nucleate pollen (Brewbaker, 1967), (5) starchy vs. starchless pollen (Baker and Baker, 1979), and (6) dry vs. wet stigmas (Heslop-Harrison and Shivanna, 1977). This analysis was carried out only with entries that included information on the most frequent PGM components (agar, sucrose, H_3_BO_3_, Ca^2+^, Mg^2+^, K^+^, and pH). The variation in the PGM ingredients in the species groups were visualized with the help of *ggplot2* package (Wickham, 2016) in the R software version 4.0.0 (R Core Team, 2020). A Kruskal–Wallis non-parametric test at a 95% confidence interval was performed to test for significant differences among PGM requirements in the different plant groups.

## Results and Discussion

### The Compendium of PGM for Pollen Research

The final version of the PGM compendium (Supplementary Appendix 1) is composed of 1572 recipes successfully used to germinate pollen grains and/or produce pollen tubes in 816 species representing 412 genera and 114 families (both monocots and dicots). All together, we recorded 110 components from the different *in vitro* PGM, used under varying conditions and concentrations. Out of 816 species, 51% (420) and 32% (260) germinated in liquid or solidified (mainly agar with concentrations 0.5–1.5%) media, respectively, while 17% (136) germinated in both solid and in liquid media. The liquid media is preferred when pollen needs to reach a certain turgescence level to germinate (Martin and Brewbaker, 1971; Montaner et al., 2003), but also because water serves other hydrolytic and synthetic reactions (Brink, 1924). However, in some species, cultivation in liquid media leads to pollen bursting, due to quick hydration thus solid (e.g., agarified) media is required (Burke, 2002; Sun et al., 2008; Jayaprakash, 2018). In such media, agar also enables incorporation of sucrose or other stimulants, helps to maintain relative humidity at constant levels and provides appropriate aerobic conditions for adequate PG (Linskens and Stanley, 1974).

Among other components, sucrose (89% of species), H_3_BO_3_ (77%), Ca^2+^ (59%), Mg^2+^ (44%), and K^+^ (39%) were the most frequently used while enzymes, vitamins, and amino acids were less common (1, 6, and 1%, respectively); PGM pH values were reported in 35% of all studies reviewed. The high frequency of the former five components in the extracted PGM correspond to other studies on PGM (e.g., Imani, 2012; Jayaprakash, 2018; Wani et al., 2020) and might reflect the wide application of the classic Brewbaker and Kwack PGM (Brewbaker and Kwack, 1963) in pollen research. As for the roles of the most frequent PGM components, sucrose serves as an effective energy source and an osmoticum for PG *in vitro* (Heslop-Harrison and Heslop-Harrison, 1992; Rodriguez-Enriquez et al., 2013; Selinski and Scheibe, 2014; Reinders, 2016). Pollen bursting and failure to germinate *in vitro* is often associated with inadequate sucrose concentrations (Baloch et al., 2001). The mineral elements boron and calcium have also been found to play several critical regulatory and structural functions in PG. For instance, boron is required for the pollen wall structure, absorption, and metabolism of sugars by forming a sugar–borate complex, and increases oxygen uptake for metabolism (Vasil, 1960; Sidhu and Malik, 1986; Yang and Li, 1999; Wang et al., 2003). Boron deficiency in PGM, often leads to pollen tube bursting or failed PTG (Cheng and Rerkasem, 1993; Fang et al., 2019).

Similarly, calcium is a central regulator, providing various governing roles in the initiation and regulation of PG through ionic balance and cell signaling (Brewbaker and Kwack, 1963; Steinhorst and Kudla, 2013). Calcium in *in vitro* PGM has proved essential in pollen tip growth (Steinhorst and Kudla, 2013) with its deficiency leading to morphological abnormalities such as coiling and tip swelling (Shivanna and Rangaswamy, 1992; Taylor and Hepler, 1997). Other combined roles of boron and calcium (e.g., in sugar synthesis and accumulation) have been emphasized in different studies (Shu-juan, 2010; Muengkaew et al., 2017). Elements such as magnesium and potassium were also frequently used ingredients in PGM because of their role in cellular physiological processes, such as osmotic balance and membrane potential (Čapková-Balatková et al., 1980; Taylor and Hepler, 1997; Song et al., 2009; Wu et al., 2011). They also improve the germination and elongation of pollen tubes by enhancing the calcium effect (Brewbaker and Kwack, 1963). The pH of the *in vitro* germination medium is an important factor controlling PG and pollen tube development in different plant species (Bellani et al., 1997; Munzuroglu et al., 2003; Burke et al., 2004; Zaman, 2011; Fragallah et al., 2019), because it affects physiological processes through enzyme activation or inhibition (Fan, 2001; Bisswanger, 2014). Finally, the comparatively low percentages of PGM components including enzymes, vitamins, and amino acids can be explained by the fact that pollen grains are rich in these components and therefore do not generally require exogenous supply of such substances in the *in vitro* PGM (Campos et al., 2008; Komosinska-Vassev et al., 2015).

### PGM Requirements in Different Plant Groups

The median concentration requirements for the frequently used ingredients varied among the plant groups with different degrees of magnitude. Because data distribution in the majority of the plant groups was skewed, we preferred median values over mean values when discussing the results.

To begin with, successful PG and PTG was observed mainly in a liquid PGM (agar = 0%, Table 1) regardless of the characteristics of the tested species, except for a few cases such as in Poaceae where agar was required for some special needs (see below). The strong dominance of liquid PGM recipes in our compendium could be also explained by high popularity of the “hanging drop” cultivation approach in pollen research (Stanley and Linskens, 1974). Furthermore, pollen of the plant families strongly represented in the compendium (e.g., Solanaceae 10% and Rosaceae 9%), prefer to germinate in liquid medium. Finally, researchers might prefer liquid PGMs over the solid ones because they are slightly more practical (i.e., no need to add agar every time you want to germinate pollen).

**TABLE 1:** The median (M), mean (μ) concentrations, standard error (SE), and *p*-values of the most frequently used ingredients in *in vitro* pollen germination media across the categories.

Group		Agar (%)				Sucrose (%)				H_3_BO_3_ (mM)				Ca^2+^ (mM)				Mg^2+^ (mM)				K^+^ (mM)				pH			
	n	M	μ	SE±	p	M	μ	SE±	P	M	μ	SE±	p	M	μ	SE±	p	M	μ	SE±	p	M	μ	SE±	p	M	μ	SE±	p
Cultivated	449	0	0.3	0.02	**<0.001**	10	13	0.27	0.94	1.6	1.6	0.05	**<0.001**	1.3	1.7	0.06	**<0.001**	0.8	1	0.03	**<0.001**	1	1	0.05	**<0.001**	6.1	6.3	0.06	**<0.001**
Wild	689	0	0.2	0.02		12	12	0.29		1.3	1.2	0.03		1.8	2.7	0.09		0.8	1	0.02		3	2.3	0.09		5.5	6.1	0.07	
Angiosperm	1092	0	0.3	0.01	0.27	12	12	0.21	**<0.001**	1.6	1.3	0.03	0.43	1.7	2.4	0.07	**0.013**	0.8	1	0.02	**0.005**	1	1.9	0.07	0.16	5.9	6.2	0.05	0.42
Gymnosperm	46	0	0.3	0.07		10	8	0.71		1.6	1.3	0.08		1.3	1.3	0.08		0.8	0.8	0.1		1	1.3	0.22		5.8	5.9	0.15	
Dicotyledon	818	0	0.2	0.01	0.27	14	13	0.25	**<0.001**	1.6	1.4	0.04	0.43	1.8	2.5	0.08	**0.01**	0.8	1	0.02	**0.005**	1.3	1.9	0.08	0.16	5.8	6.2	0.07	0.42
Monocotyledon	274	0	0.4	0.03		10	12	0.38		1.6	1.3	0.04		1.3	2	0.11		0.8	1	0.03		1	1.8	0.17		6.3	6.1	0.08	
Binucleate	913	0	0.3	0.01	0.16	10	12	0.22	**<0.001**	1.6	1.4	0.03	0.23	1.7	2.4	0.07	**0.01**	0.8	1	0.02	**0.004**	2.2	1.9	0.08	0.08	5.9	6.1	0.06	0.59
Trinucleate	173	0	0.3	0.04		15	15	0.59		1.6	1.3	0.05		1.7	2.3	0.14		1	1	0.03		1	1.7	0.21		5.8	6.5	0.14	
Dry	604	0	0.3	0.02	0.2	15	12	0.3	**<0.001**	1.6	1.3	0.04	0.43	1.8	2.6	0.09	**0.004**	0.8	1	0.02	**0.001**	3	2.2	0.1	0.06	5.8	6.3	0.08	0.58
Wet	475	0	0.3	0.02		10	12	0.29		1.6	1.4	0.05		1.3	2.1	0.09		0.8	1	0.02		1	1.6	0.1		6.0	6.1	0.06	
Starchless	268	0	0.3	0.03	0.47	10	12	0.41	0.19	1.6	1.3	0.05	0.99	1.3	2.2	0.13	0.92	0.8	1	0.03	0.39	2.6	2	0.16	**0.02**	6.2	6.1	0.09	0.55
Starchy	86	0	0.1	0.04		10	11	0.79		1.6	1.4	0.11		1.8	3.1	0.25		1	1.1	0.05		3	2.5	0.2		5.5	6.0	0.21	

*Bold values indicate statistically significant results.*

Among other components considered, pollen requirements for H_3_BO_3_ (1.6 mM) and pH (range 5.5–6.3) in PGM were similar across all plant groups, except for species with different cultivation status (CW; Table 1). The former being similar could be attributed to the broad usage of Brewbaker–Kwack protocol in our dataset, whose boric acid concentration has proved to be right in many studies (Chauhan et al., 1987; Kumari et al., 2015; Souza et al., 2017). Correspondingly, boric acid is the least variable component in PGMs, markedly affecting PG and PTG (Stanley, 1971) with small deviations making it either inadequate (Cheng and Rerkasem, 1993; Fang et al., 2019) or toxic (Fang et al., 2016). Similarly, slight or drastic changes in the pH media can affect the pollen cytoplasmic pH resulting in slow pollen growth or total growth inhibition (Tupý and Řhová, 1984; Fricker et al., 1997; Fan, 2001). This suggests that boron has equal importance in PG and PTG regardless of the pollen anatomy, morphology, and physiology.

As for the remaining most frequently used PGM components, Ca^2+^ and Mg^2+^ were significantly different in all categories except for species with starch vs. starchless pollen (SS). Sucrose concentration was also significantly different in all groups compared, except for SS and CW (Table 1). Notably, although Mg^2+^ was statistically significant in all categories (except SS), the differences in both median and mean values were marginal (Mg^2+^ median concentration range: 0.8–1 mM; Table 1). This can be explained by low data variability in our dataset (Supplementary Appendix 1), affected by wide usage of PGM based on the Brewbaker and Kwack PG protocol (Brewbaker and Kwack, 1963). The group-specific difference and possible underlying reasons are discussed in the following paragraphs.

**FIGURE 1:**
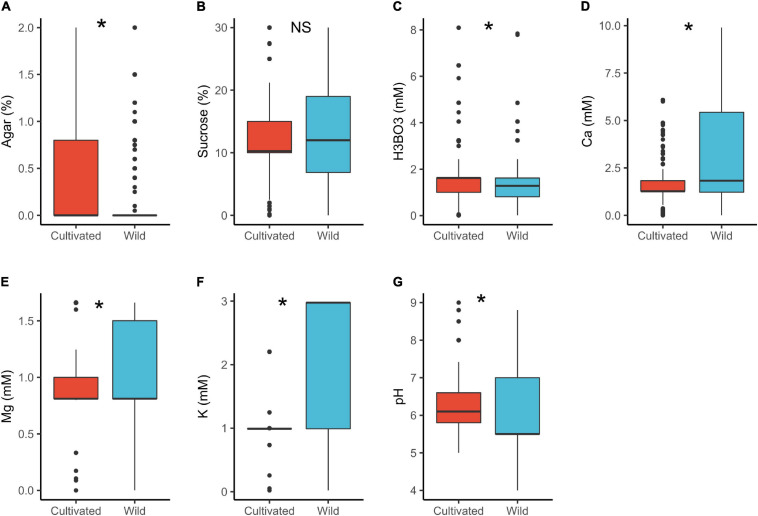
Pollen germination media requirements for cultivated (*n* = 449) and wild species (*n* = 689): agar **(A)**, sucrose **(B)**, boric acid **(C)**, calcium **(D)**, magnesium **(E)**, potassium **(F)**, and pH **(G)**. *, significant (*p* < 0.05); NS, not significant.

In cultivated plants, the median concentration of H_3_BO_3_ (1.6 mM) and pH (6.1) were significantly higher than in wild plants (H_3_BO_3_ = 1.3 mM and pH = 5.5). In contrast, wild species had significantly higher concentrations of Ca^2+^ (1.8 mM) and K^+^ (3.0 mM) than the cultivated ones (Ca^2+^ = 1.3 mM, K^+^ = 1.0 mM; Table 1 and Figure 1). Some studies indicate that pollen of wild species have lower PGM requirements than the cultivated ones (Jayaprakash, 2018) as was observed in the case of boric acid. However, we have no a plausible explanation for the pH differences or why the mineral concentrations were higher in the wild plants than the cultivated. The differences could be attributed to domestication and artificial selection, which may cause cultivars to change their physiological and morphological attributes thus differing from the wild counterparts (Chaudhary, 2013; Gepts, 2014; Liu et al., 2019). Finally, the experimental design and selection of various conditions for domesticated and wild species by different researchers could as well have contributed to the observed differences.

**FIGURE 2:**
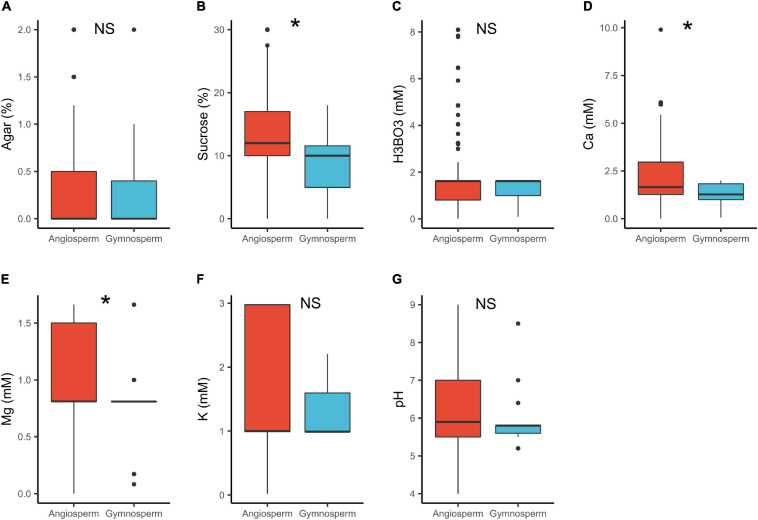
Pollen germination media requirements for angiosperms (*n* = 1092) and gymnosperms (*n* = 46): agar **(A)**, sucrose **(B)**, boric acid **(C)**, calcium **(D)**, magnesium **(E)**, potassium **(F)**, and pH **(G)**. *, significant (*p* < 0.05); NS, not significant.

In angiosperms, the median concentrations of sucrose (12%) and Ca^2+^ (1.7 mM) were significantly higher than in gymnosperms (sucrose = 10%, Ca^2+^ = 1.3 mM; Table 1 and Figure 2). These results are in line with the study by Brewbaker and Kwack (1963) that shows that gymnosperm pollen generally require lower concentration of these substances (Brewbaker and Kwack, 1963). The lower calcium requirements can be attributed to the generally comparatively slow growth of germinating gymnosperm pollen (Brewbaker and Kwack, 1963; Williams, 2012). The dissimilarity in the cytology and wall structure between angiosperms and gymnosperms pollen (e.g., Pacini et al., 1999; Fernando et al., 2005) could probably also contribute to the observed differences. Further, pollen cytology plays a significant role in the metabolic processes (Bergamini and Mulcahy, 1988). For instance, pollen grains of most gymnosperms are wind pollinated (Faegri and van der Pijl, 1979) and hence, released with a low moisture content to reduce weight (Fernando et al., 2005). In order for gymnosperm pollen to germinate, rehydration takes place quickly in the liquid medium/pollination drop within the micropylar (Nepi et al., 2005; Firon et al., 2012). This suggests that a higher osmotic potential (lower salt content) is required probably explaining the lower concentrations of sucrose and Ca^2+^ observed in the PGM for gymnosperms. Contrary, angiosperm pollen is released with higher moisture content with rehydration being slower, and grows hurriedly, utilizing the available ingredients quickly (Dawkins and Owens, 1993; Pacini et al., 1999; Nepi et al., 2005; Firon et al., 2012). This probably explains the higher concentration of sucrose and Ca^2+^ in the *in vitro* media.

**FIGURE 3:**
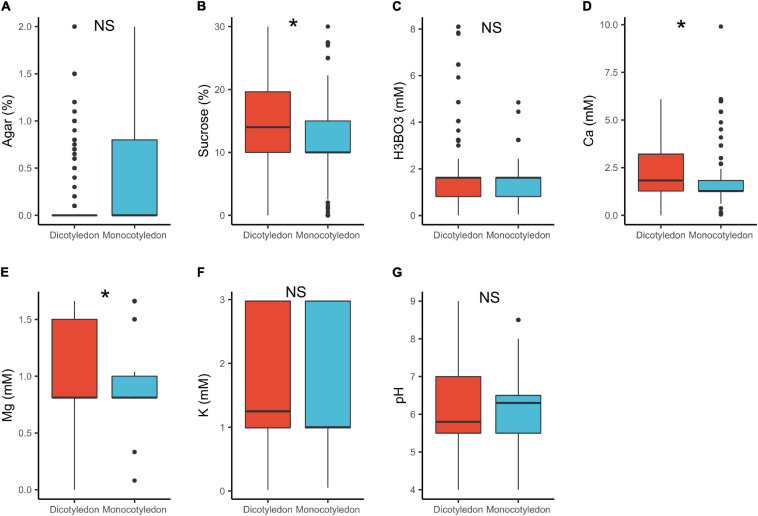
Pollen germination media requirements for dicotyledons (*n* = 818) and monocotyledons (*n* = 274): agar **(A)**, sucrose **(B)**, boric acid **(C)**, calcium **(D)**, magnesium **(E)**, potassium **(F)**, and pH **(G)**. *, significant (*p* < 0.05); NS, not significant.

In dicots, the median concentrations of sucrose (14%) and Ca^2+^ (1.8 mM) were significantly higher, than in monocots (sucrose = 10%, Ca^2+^ = 1.3 mM; Table 1 and Figure 3). These results are in accordance with previous research showing that monocot pollen require lower mineral content than dicots (Jayaprakash, 2018). A possible explanation is that monocot pollen is usually recalcitrant (i.e., have high moisture content of 30–40%) as compared to orthodox pollen of dicots (1–5%; Franchi et al., 2011; Jayaprakash, 2018). This suggests therefore, that monocot pollen requires lower ion concentrations in a PGM to reach the turgence level needed to germinate. Additionally, pollen of several monocots such as *Typha latifolia* L., are released with high concentration of sugars in it (mainly sucrose) and other protective molecules to confer stability after release, an adaptation against desiccation (Wolkers et al., 2001; Pacini et al., 2006). They therefore require low concentrations of these in the media. Contrastingly, the largely orthodox dicot pollen are desiccation tolerant and are released with low moisture and mineral content (Wolkers et al., 2001; Pacini et al., 2006; Franchi et al., 2011), hence requiring a higher supply of these substances in *in vitro* media.

**FIGURE 4:**
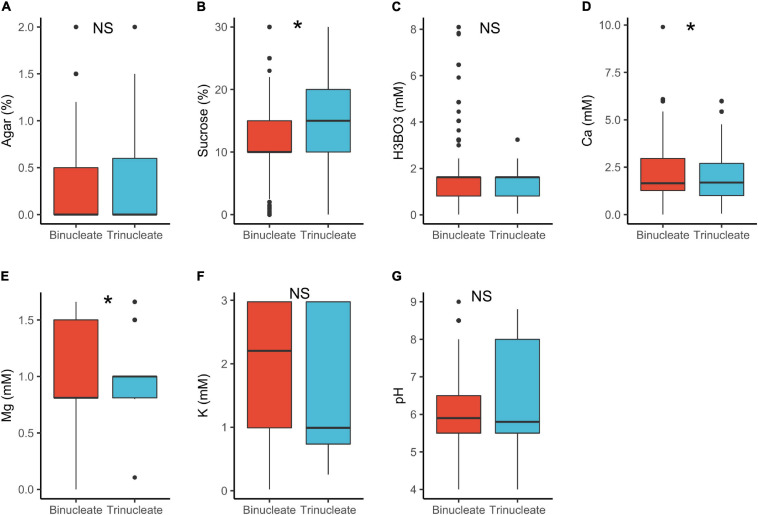
Pollen germination media requirements for binucleate (*n* = 913) and trinucleate pollen (*n* = 713): agar **(A)**, sucrose **(B)**, boric acid **(C)**, calcium **(D)**, magnesium **(E)**, potassium **(F)**, and pH **(G)**. *, significant (*p* < 0.05); NS, not significant.

In tri-nucleated pollen, requirements for sucrose in PGM were significantly higher than in binucleated pollen (15 vs. 10%, respectively; Table 1 and Figure 4). The component requirements *in vitro* highly depend on the existing endogenous pollen reserves, whether these are sustainable autotrophically and are readily available for metabolism (Read et al., 1993; Stephenson et al., 2003; Carrizo García et al., 2012). Trinucleated pollen is found to be highly dependent on exogeneous supply of substances important for PG and PTG (Mulcahy and Mulcahy, 1988). This can be associated with tricellular pollen germinating faster, a heterochronic evolutionary shift from bicellular pollen (Brewbaker, 1967; Mulcahy and Mulcahy, 1988), accounting for the higher sucrose requirements observed. In contrast, binucleated pollen grains initially rely on their own reserves when germinating *in vitro*, hence the lower sucrose requirements (Mulcahy and Mulcahy, 1988). Pollen requirements for Ca^2+^ in PGM were also statistically different between tri- vs. bi-nucleated pollen but the median values were similar (1.7 mM; Table 1) as was the case for Mg^2+^.

**FIGURE 5:**
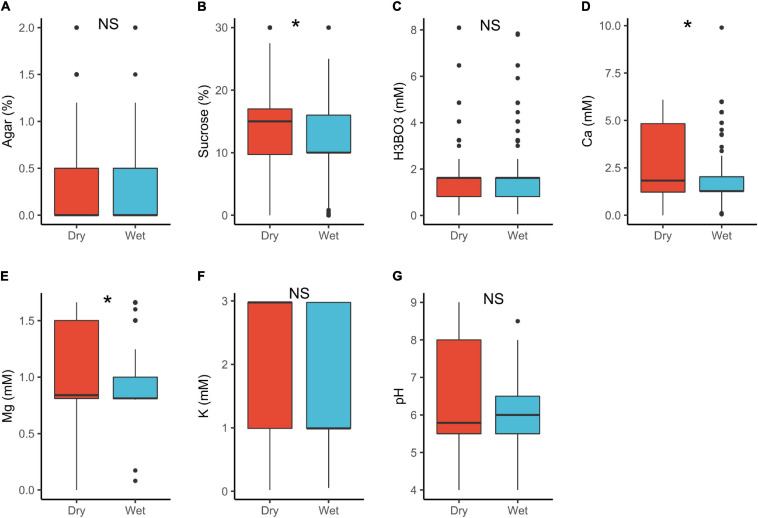
Pollen germination media requirements for dry stigma (*n* = 604) and wet stigma plants (*n* = 475): agar **(A)**, sucrose **(B)**, boric acid **(C)**, calcium **(D)**, magnesium **(E)**, potassium **(F)**, and pH **(G)**. *, significant (*p* < 0.05); NS, not significant.

In plant with dry stigmas, sucrose (15%) and Ca^2+^ (1.8 mM) requirements were significantly higher than in wet stigma plants (sucrose = 10%, Ca^2+^ = 1.3 mM; Table 1 and Figure 5). Under natural conditions, wet stigmatic plants produce exudates rich in minerals and sugars (Labarca et al., 1970; Hawker et al., 1983; Kandasamy and Vivekanandan, 1983; Lau et al., 2017) in which pollen germinates (Park and Lord, 2003; Allen and Hiscock, 2010; Rejón et al., 2014). This would suggest that pollen in wet stigmatic plants should as well require a higher exogenous supply of these substances *in vitro*. However, this was contrary with our results. A possible explanation is that most dry stigmatic plants tend to have trinucleate pollen with high requirements for exogenous minerals (see also above), whereas wet stigmas are associated with binucleated pollen (lower supply; Brewbaker, 1967; Montaner et al., 2003).

**FIGURE 6:**
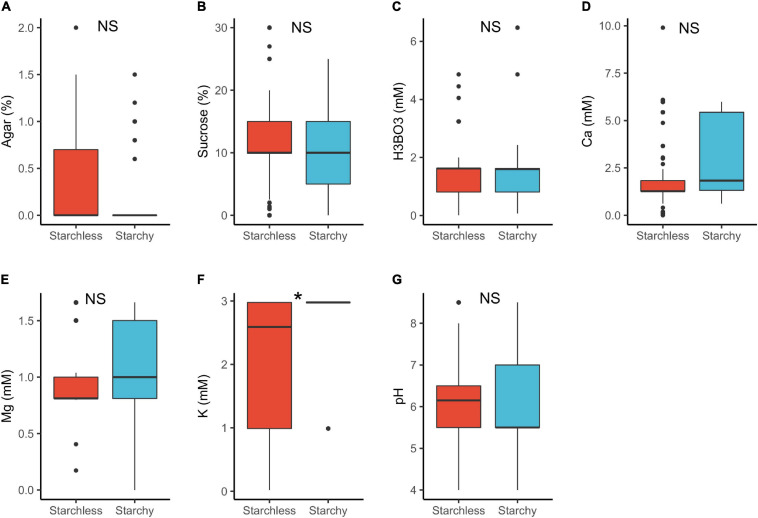
Pollen germination media requirements for starchy (*n* = 86) and starchless pollen (*n* = 268): agar **(A)**, sucrose **(B)**, boric acid **(C)**, calcium **(D)**, magnesium **(E)**, potassium **(F)**, and pH **(G)**. *, significant (*p* < 0.05); NS, not significant.

In plants with starchy vs. starchless pollen, we detected statistically significant differences for K^+^ requirements only (Table 1 and Figure 6). The lower requirements of starchless pollen (median 2.6 mM) than in starchy (3.0 mM) can be attributed to the fact that the former are usually associated to binucleated pollen which require lower mineral input than in starchy/trinucleate (Lora et al., 2012). Notably, both pollen types can occur in the same anther (Baker and Baker, 1979; Lora et al., 2012) and yet may need different requirements to germinate *in vitro* (Franchi et al., 2007).

As for the groupings by taxonomy, plant families Solanaceae (10%), Rosaceae (9%), Fabaceae (8%), Poaceae and Pinaceae at 5% had the strongest representation in the compendium accounting all together for 586 PGM recipes for 285 (35%) species. The high contribution from these five families can be explained by the fact that many species from these families are cultivated plants (e.g., Solanaceae: tomato, potato; Rosaceae: almond, apple, pear, cherry; Fabaceae: peas and beans; Poaceae: rice, wheat, barely; Pinaceae: pines). *Nicotiana tabacum* L. (27 recipes), *Zea mays* L. (25), and *Lilium longiflorum* Thunb. (22), which are also economically (*Z. mays*) and scientifically (*N. tabacum* and *L. longiflorum*) important plants, were the most studied species. *N. tabacum* L., the tobacco plant, has also been widely used as a model plant in pollen research, as it produces long-living pollen in large quantities (Cheung et al., 1995; Conze et al., 2017). The medium requirements for the most frequently cultivated species as well as model species such as *A. thaliana* (L.) Heynh. are summarized in the Table 2.

**TABLE 2:** The median (M), mean (μ) concentrations, and standard error of the most frequently used ingredients in *in vitro* pollen germination media for the most studied species.

Species		Agar (%)			Sucrose (%)			H_3_BO_3_ (mM)			Ca^2+^ (mM)			Mg^2+^ (mM)			K^+^ (mM)			pH		
	n	M	μ	SE±	M	μ	SE±	M	μ	SE±	M	μ	SE±	M	μ	SE±	M	μ	SE±	M	μ	SE±
*Arabidopsis thaliana* (L.) Heynh.	6	0.6	0.6	0.16	18	16	2.72	1.6	1.5	0.16	4	2.9	0.93	1	0.7	0.3	0.6	0.6	0.37	7	6.9	0.25
*Lilium longiflorum* Thunb.	19	0	0.1	0.07	10	9	1.05	0.2	0.8	0.16	1.3	1.7	0.64	0.9	0.9	0.09	1	1	0	5.6	5.5	0.11
*Lotus corniculatus* L.	11	0	0.1	0.09	0	6	2.6	0.8	1	0.11	5.4	4.8	0.53	1.2	1.2	0.12	3	2.6	0.37	5.5	5.9	0.44
*Lycopersicon esculentum* Mill.	12	0	0.5	0.19	12	12	1.78	1	1.2	0.18	1.3	1.6	0.35	0.8	1	0.21	–	–	–	6	6.1	0.37
*Nicotiana tabacum* L.	23	0	0	0.03	10	10	0.9	1.6	1.6	0.13	1.3	1.6	0.25	0.8	1	0.08	1	1	0	6	6.3	0.14
*Olea europaea* L.	8	0.5	0.4	0.12	10	12	1.38	1.6	2.5	0.71	1.5	1.5	0.28	1.2	1.2	0.43	1	1	–	5.3	5.4	0.23
*Petunia hybrida* Vilm.	13	0	0.1	0.09	10	11	1.15	1.6	1.6	0.17	1.3	1.7	0.69	0.8	1.1	0.28	1	1	–	6	6.0	0.07
*Prunus dulcis* (Mill.) D.A.Webb	5	1	0.6	0.23	15	13	1.22	1.6	1.3	0.31	1.5	1.5	0.28	0.8	0.8	–	1	1	–	5.8	5.8	–
*Zea mays* L.	16	0.6	0.6	0.11	15	15	0.51	1.6	1.3	0.15	1.8	1.8	0.14	–	–	–	–	–	–	6	6.1	0.29

The PGM requirements for the most used components also tend to be different for the families. Among the frequent families, only Poaceae pollen tends to germinate better and produce pollen tubes on solid medium (0.6% agar; Table 3) with the rest of the families preferring liquid medium. The tendency of Poaceae pollen to germinate in PGM with higher agar concentration can be explained by the dry stigmata in this family. Germinating pollen of plants with dry stigmas has often proved challenging as it is more difficult to imitate the complex interactions on dry stigmatic surfaces (Allen and Hiscock, 2010; Rodriguez-Enriquez et al., 2013). Therefore, the higher agar concentration in PGM of pollen from dry stigmatic plants helps in incorporating the ingredients, maintaining humidity, and adequate aerobic conditions for PG (Linskens and Stanley, 1974). In contrast, the rest of frequent families mostly have wet stigmas with exudates (Allen and Hiscock, 2010) which are easier to imitate *in vitro* and germinate the pollen in liquid media.

**TABLE 3:** The median (M), mean (μ) concentrations, and standard error of the most frequently used ingredients in *in vitro* pollen germination media for the most represented families.

Family		Agar (%)			Sucrose (%)			H_3_BO_3_ (mM)			Ca^2+^ (mM)			Mg^2+^ (mM)			K^+^ (mM)			pH		
	n	M	μ	SE±	M	μ	SE±	M	μ	SE±	M	μ	SE±	M	μ	SE±	M	μ	SE±	*M*	μ	SE±
Fabaceae	90	0	0.1	0.04	10	12	0.94	1.6	1.3	0.07	1.8	3.1	0.23	0.8	1.1	0.05	2.6	2	0.19	5.5	5.91	0.18
Liliaceae*	58	0	0.2	0.05	10	9	0.63	1.6	1.1	0.09	1.8	2.7	0.33	1	1.2	0.09	2	1.9	0.43	5.7	5.98	0.20
Pinaceae	38	0	0.3	0.07	9	8	0.78	1.6	1.3	0.09	1.3	1.3	0.09	0.8	0.7	0.12	1	1.4	0.26	5.8	5.72	0.06
Poaceae	59	0.6	0.6	0.07	15	16	0.99	1.6	1.3	0.1	1.5	1.8	0.17	0.9	1.1	0.17	0.7	0.7	0	5.8	5.88	0.08
Rosaceae	87	0	0.4	0.05	10	12	0.52	1.6	1.5	0.16	1.3	1.7	0.17	0.8	1	0.05	1	1.6	0.37	6	6.12	0.14
Solanaceae	139	0	0.2	0.03	15	14	0.54	1	1.4	0.09	1.3	1.5	0.14	0.8	1	0.06	1	1.1	0.08	6	6.03	0.08

*Includes species from other families such as Amaryllidaceae.

Furthermore, Poaceae and Solanaceae tend to have comparatively higher sucrose requirements than the other frequent families (15 vs. 9–10%; Table 3). Notably, some Poaceae species such as *Triticum aestivum* L. even require higher concentrations of other sugars such as maltose (18–30%; Supplementary Appendix 1). This can be explained by Poaceae having trinucleated pollen that mostly rely on the exogenous mineral supply (also see above). Solanaceae having high sucrose requirements can be attributed to the high data variability in our dataset for this family (sucrose concentration range 10–20%). Generally, pollen anatomy, morphology, and physiology can vary considerably among species (Baker and Baker, 1979; Pacini, 1996; Speranza et al., 1997) contributing to observed differences in the media requirements among families (Table 3).

### Developing PGM Recipes

The detected (dis)similarities in pollen requirements for the most frequently used PGM components can be used for creating a new PGM where no ready recipe is available in our data set. Below, we suggest a number of “base” recipes for various plant groups:

• Angiosperms: liquid media, 12% sucrose, 1.6 mM H_3_BO_3_, 1.7 mM Ca^2+^, 0.8 mM Mg^2+^, 1.0 mM K^+^, and pH 5.9
• Gymnosperms: liquid media, 10% sucrose, 1.6 mM H_3_BO_3_, 1.3 mM Ca^2+^, 0.8 mM Mg^2+^, 1.0 mM K^+^, and pH 5.8
• Dicots: liquid media, 14% sucrose, 1.6 mM H_3_BO_3_, 1.8 mM Ca^2+^, 0.8 mM Mg^2+^, 1.3 mM K^+^, and pH 5.8
• Monocots: liquid media, 10% sucrose, 1.6 mM H_3_BO_3_, 1.3 mM Ca^2+^, 0.8 mM Mg^2+^, 1.0 mM K^+^, and pH 6.3
• Binucleate pollen: liquid media, 10% sucrose, 1.6 mM H_3_BO_3_, 1.7 mM Ca^2+^, 0.8 mM Mg^2+^, 2.2 mM K^+^, and pH 5.9
• Trinucleate pollen: liquid media, 15% sucrose, 1.6 mM H_3_BO_3_, 1.7 mM Ca^2+^, 1.0 mM Mg^2+^, 1.0 mM K^+^, and pH 5.8
• Dry stigmatic plants: liquid media, 15% sucrose, 1.6 mM H_3_BO_3_, 1.8 mM Ca^2+^, 0.8 mM Mg^2+^, 3.0 mM K^+^, and pH 5.8
• Wet stigmatic plants: liquid media, 10% sucrose, 1.6 mM H_3_BO_3_, 1.3 mM Ca^2+^, 0.8 mM Mg^2+^, 1.0 mM K^+^, and pH 6.0
• Starchless pollen: liquid media, 10% sucrose, 1.6 mM H_3_BO_3_, 1.3 mM Ca^2+^, 0.8 mM Mg^2+^, 2.6 mM K^+^, and pH 6.2

• Starchy pollen: liquid media, 10% sucrose, 1.6 mM H_3_BO_3_, 1.8 mM Ca^2+^, 1.0 mM Mg^2+^, 3.0 mM K^+^, and pH 5.5.

For some frequent families, the following from our data set can be used as a guide in creating new PGM:

• Fabaceae: liquid media, 10% sucrose, 1.6 mM H_3_BO_3_, 1.8 mM Ca^2+^, 0.8 mM Mg^2+^, 2.6 mM K^+^, and pH 5.5
• Pinaceae: liquid media, 9% sucrose, 1.6 mM H_3_BO_3_, 1.3 mM Ca^2+^, 0.8 mM Mg^2+^, 1.0 mM K^+^, and pH 5.8
• Poaceae: 0.6% agar, 15% sucrose, 1.6 mM H_3_BO_3_, 1.5 mM Ca^2+^, 0.9 mM Mg^2+^, 0.7 mM K^+^, and pH 5.8
• Rosaceae: liquid media, 10% sucrose, 1.6 mM H_3_BO_3_, 1.3 mM Ca^2+^, 0.8 mM Mg^2+^, 1.0 mM K^+^, and pH 6
• Solanaceae: liquid media, 15% sucrose, 1.0 mM H_3_BO_3_, 1.3 mM Ca^2+^, 0.8 mM Mg^2+^, 1.0 mM K^+^, and pH 6.

## Data Availability Statement

The original contributions presented in the study are included in the article/Supplementary Material, further inquiries can be directed to the corresponding author/s.

## Author Contributions

SR designed the study. DT and SR made substantial contributions to the acquisition, analysis, and interpretation of the work. Both authors proofed and corrected the manuscript.

## Conflict of Interest

The authors declare that the research was conducted in the absence of any commercial or financial relationships that could be construed as a potential conflict of interest.

## Footnotes

1 www.webofknowledge.com

## Supplementary Material

Supplementary Appendix 1 Compendium of pollen germination media.
